# Assessing nucleic acid binding activity of four dinoflagellate cold shock domain proteins from *Symbiodinium kawagutii and Lingulodinium polyedra*

**DOI:** 10.1186/s12860-021-00368-4

**Published:** 2021-05-08

**Authors:** Bahareh Zaheri, David Morse

**Affiliations:** Institut de Recherche en Biologie Végétale, Département de Sciences Biologiques, 4101 Sherbrooke Est, Université de Montréal, Montréal, H1X 2B2 Canada

**Keywords:** Transcription factors, Cold shock domain proteins, Dinoflagellates, RNA binding domain, DNA binding domain, Transcription

## Abstract

**Background:**

Dinoflagellates have a generally large number of genes but only a small percentage of these are annotated as transcription factors. Cold shock domain (CSD) containing proteins (CSPs) account for roughly 60% of these. CSDs are not prevalent in other eukaryotic lineages, perhaps suggesting a lineage-specific expansion of this type of transcription factors in dinoflagellates, but there is little experimental data to support a role for dinoflagellate CSPs as transcription factors. Here we evaluate the hypothesis that dinoflagellate CSPs can act as transcription factors by binding double-stranded DNA in a sequence dependent manner.

**Results:**

We find that both electrophoretic mobility shift assay (EMSA) competition experiments and selection and amplification binding (SAAB) assays indicate binding is not sequence specific for four different CSPs from two dinoflagellate species. Competition experiments indicate all four CSPs bind to RNA better than double-stranded DNA.

**Conclusion:**

Dinoflagellate CSPs do not share the nucleic acid binding properties expected for them to function as *bone fide* transcription factors. We conclude the transcription factor complement of dinoflagellates is even smaller than previously thought suggesting that dinoflagellates have a reduced dependance on transcriptional control compared to other eukaryotes.

**Supplementary Information:**

The online version contains supplementary material available at 10.1186/s12860-021-00368-4.

## Background

Dinoflagellates are an important group of unicellular eukaryotes perhaps best known for their large genomes and permanently condensed chromosomes. Surprisingly, little is known how gene expression is regulated in these organisms. Transcriptome analyses in several species, including *Lingulodinium* and *Symbiodinium,* have revealed a general paucity (typically 0.15%) of sequences annotated as transcription factors (TF). This is in sharp contrast to the roughly 6% of genes annotated as TF in plants [1] or animals [2]. In addition, a high proportion (~ 60%) of the annotated dinoflagellate TF in transcriptomes are cold shock domain (CSD) containing proteins (CSPs) [3, 4] yet this class is typically less than 1% of the TF in other eucaryotes. CSDs are small (roughly 70 amino acid) nucleic acid binding domains containing two conserved RNA recognition motifs**,** KGFGFI and VFVHF, that are known to bind both DNA and RNA. All dinoflagellate CSPs contain the two RNA binding motifs characteristic of the CSD. Four divergent domain structures have been found in *Lingulodinium* and *Symbiodinium* proteins, the most prevalent ones containing a CSD either alone or with a C-terminal G-rich domain. Less frequently observed are some structures containing a Zn-finger domain following the G-rich domain, and also examples of sequences with multiple CSDs and one or more RNA recognition motifs (RRM). Thus, many of the dinoflagellate CSPs are similar to what are found in bacteria as these typically contain only a CSD [5].

In *E. coli*, CSPs have a wide range of functions, including binding DNA as transcription factors, binding to RNA, regulating transcription, splicing, and translation, and affecting mRNA stability as RNA chaperones [6, 7]. Bacterial CSPs have a non-specific RNA binding function during cold stress, which is correlated to their chaperone activity, and this helps transcription by acting as an antiterminator [7, 8]. However, the dinoflagellate proteins may be different from their bacterial counterparts as two *Lingulodinium* CSPs, both containing a single CSD followed by a glycine-rich C-terminal region, were both unable to complement the growth of an *E. coli* strain lacking four different CSP genes at low temperature [5]. Furthermore, cold temperatures did not induce the CSP transcripts in *L. polyedra* [9]. Previous work on *L. polyedra* CSPs showed binding to both single- and double-stranded DNA as well as to RNA, but it was unclear if binding would show any sequence specificity that would be likely if they were to function as transcription factors [5]. Here we performed two experimental approaches to assess the specific nucleic acid binding activity of *L. polyedra* CSP1 (*Lp*CSP1*)* and three *S. kawagutii* CSPs *(Sk*CSP1, *Sk*CSP2 and *Sk*CSP3*)*. Initially, these four CSPs were expressed, purified and used in electrophoretic mobility assays (EMSAs) to measure if they were active in binding nucleic acids. In a second approach, selection and amplification binding assays (SAAB) was used to determine if these proteins could bind a specific sequence on DNA. All these CSPs were able to bind to DNA and RNA, and no sequence specific binding activity toward DNA was observed.

## Results

### *Sk*CSP1, *Sk*CSP2 and *Sk*CSP3 belong to a *Symbiodinium* unique clade

**Fig. 1:**
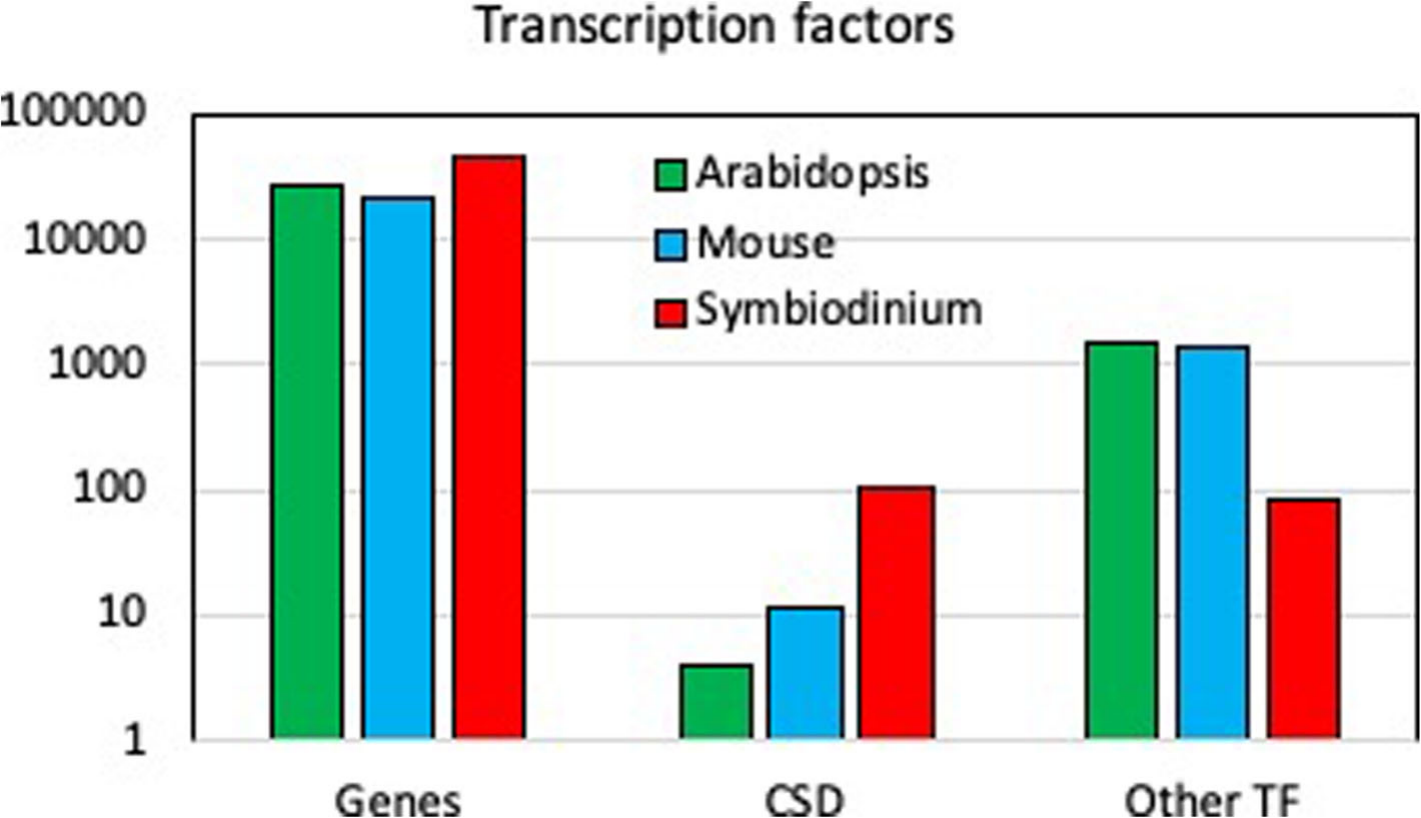
The abundance of DNA-binding domain families detected in *S. kawagutii* compared with plants and animals. The number of genes annotated as CSD and as other TF are shown for the most recent *S. kawagutii* genome. Note the log scale at left

**Fig. 2:**
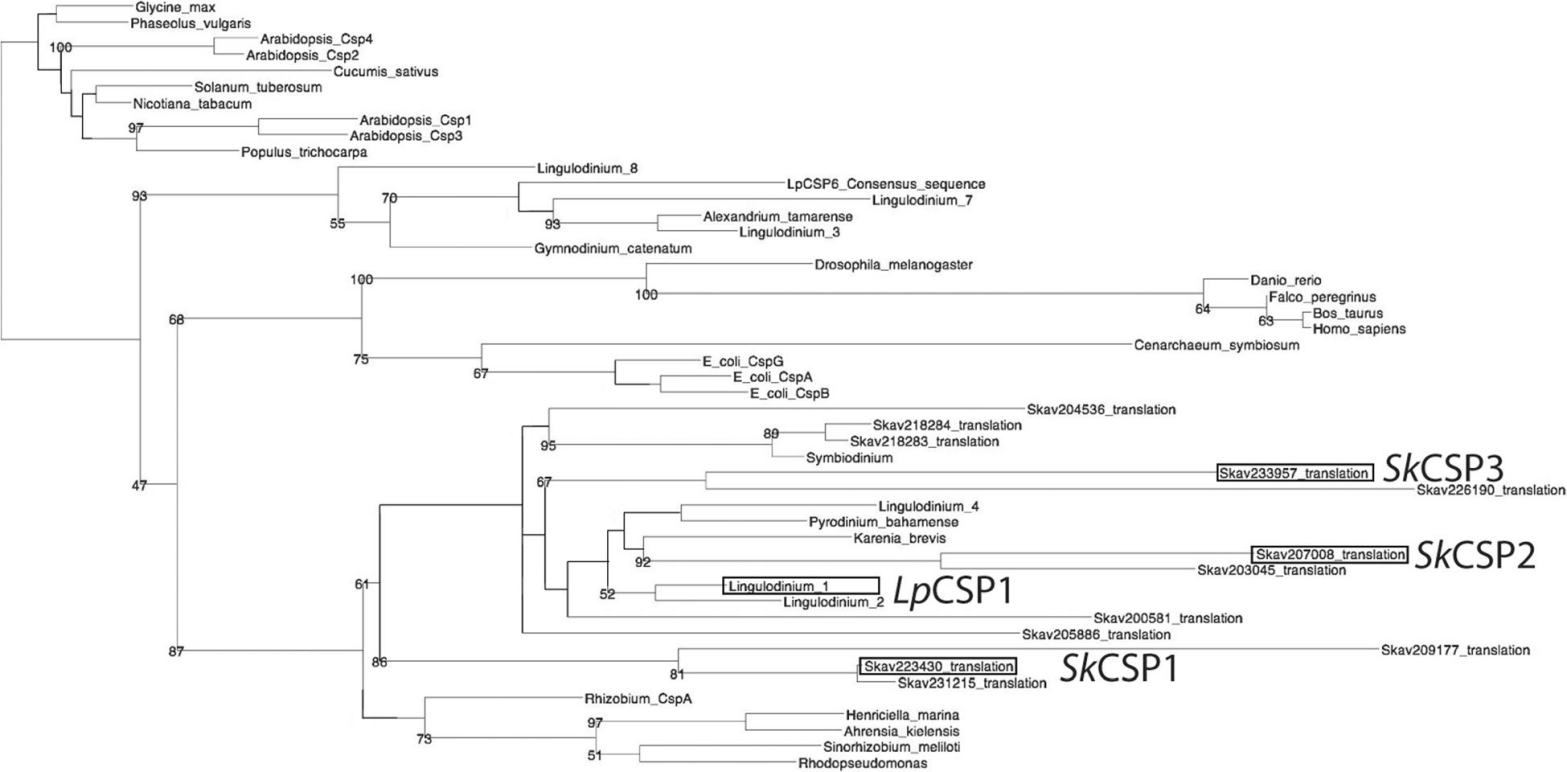
Phylogenetic reconstruction of a variety of dinoflagellate CSP. Sequences were aligned and the phylogeny reconstructed with RaxML. *Lp*CSP1*, Sk*CSP1, *Sk*CSP2 and *Sk*CSP3 sequences are boxed

The number of annotated DNA binding proteins in the genome of the *S. kawagutii* genome [10] belonging either to CSD family or other TF (Fig. 1) shows the relative importance of CSDs in dinoflagellates compared to plants and animals. All CSDs contain the two RNA recognition motifs (KGFGFI and VFVHF) shared with bacteria and plants [3, 4]. Phylogenetic analysis of CSDs from 12 predicted *Symbiodinium kawagutii* protein sequences was performed using RaxML, and all were found to cluster together within a single well defined clade together with some bacterial sequences (Fig. 2, Table S2). This is slightly different from the situation in *Lingulodinium* where sequences are distributed among two different clades. The phylogenetic positions of the four CSPs examined here: *Lp*CSP1 (JO732587), *Sk*CSP1 (Skav223430), *Sk*CSP2 (Skav207008) and *Sk*CSP3 (Skav233957) are boxed.

**Fig. 3:**
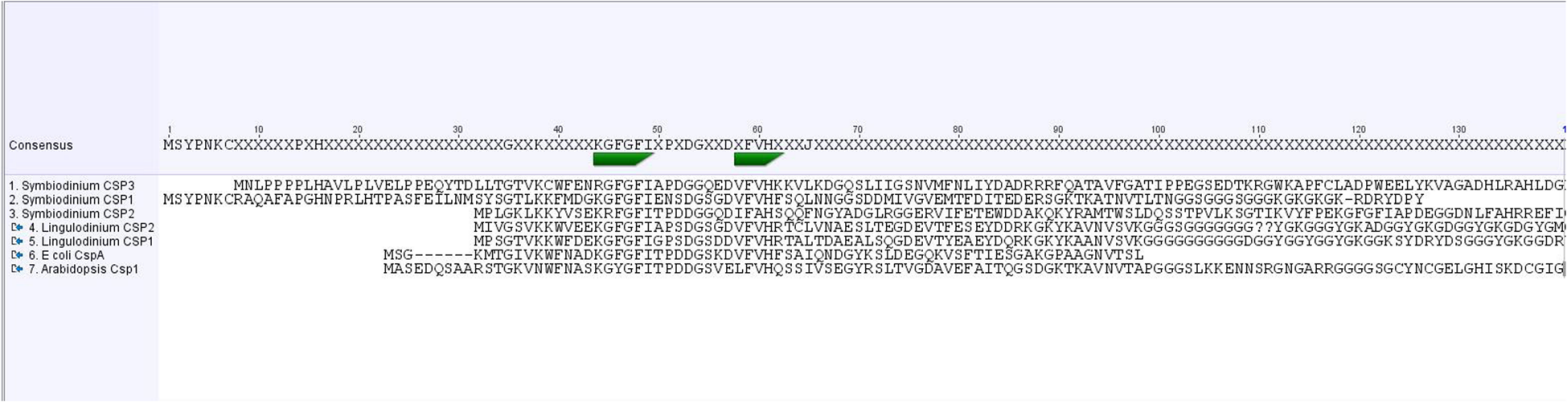
Alignment of CSD domains from the dinoflagellates *L. polyedra*, and *S. kawagutii*, the bacterium *E. coli* and the higher plant *Arabidopsis thaliana*. The two RNA recognition motifs are marked in green

*Lp*CSP1 with a size of 113 amino acids has been previously cloned [5]. For this study, *Sk*CSP1, *Sk*CSP2 and *Sk*CSP3 were also cloned and have sizes of 128, 120 and 182 residues, respectively. All four CSPs were expressed as GST-tagged proteins and used for EMSA after removal of the GST tag (Fig. S1). Two of the *S. kawagutii* proteins contain an N-terminal extension (Fig. 3).

### *Lingulodinium* and *Symbiodinium* CSPs bind to DNA and RNA

**Fig. 4:**
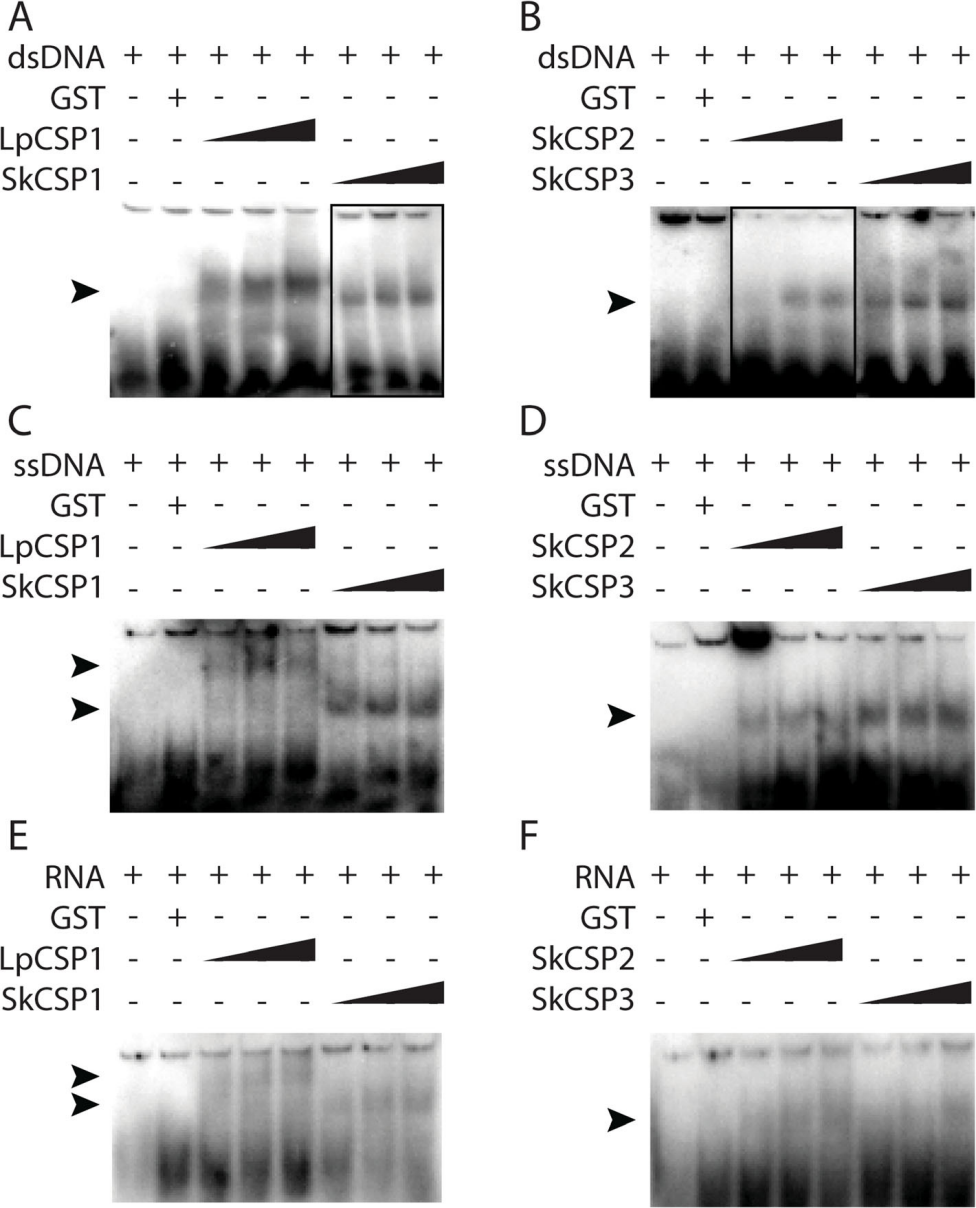
Nucleic acid binding activity of *L. polyedra* and *S. kawagutii* CSPs in EMSA. ssDNA (**a**, **b**), dsDNA (**c**, **d**) and RNA (**e**, **f**) probes were used. The black triangle shows the different concentrations of the CSPs (0.5, 1 and 3 μg in all the assays); position of the shifts are shown by arrows

EMSA experiments were conducted on *Lp*CSP1, *Sk*CSP1, *Sk*CSP2 and *Sk*CSP3 to analyze their binding to radiolabeled double-stranded (dsDNA), single-stranded (ssDNA) and RNA probes (Fig. 4). Fusion proteins still containing the glutathione *S*-transferase (GST) tags also bind nucleic acids but migrating slower on the gel, and all EMSA experiments used proteins after removal of the tag by thrombin.

All proteins were able to bind dsDNA, ssDNA and RNA as seen by the presence of a radioactive band of lower mobility. The mobility of probe sequence was reduced to roughly the same extent with all proteins with the exception of *Lp*CSP1 binding to ssDNA or RNA. The amount of the reduced mobility band seemed to increase with increasing concentrations of the CSPs, although not precisely proportional to the amount of protein. We conclude that all four CSPs were able to bind to all three types of nucleic acids tested.

### *Symbiodinium* CSP1 shows preferential binding to single-stranded nucleic acids

**Fig. 5:**
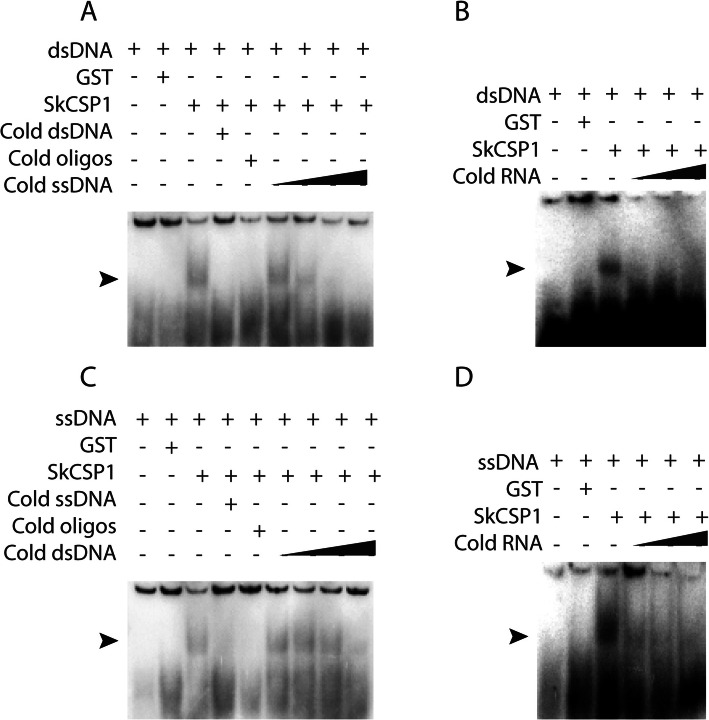
Competition assays of *SkCSP1* with ssDNA, dsDNA and RNA. *Symbiodinium* CSP1 binds to ssDNA better than dsDNA. Cold oligos have a different sequence than the ssDNA. Concentration of *Sk*CSP1 is 0.5 *μ*g in all the assays; position of the shifts are shown by arrows. RNA competes efficiently with both dsDNA and ssDNA. The black triangle shows the different concentrations of the unlabeled nucleic acids (1x, 10x, 30x, and 80x the probe concentration)

To assess the specificity of *Symbiodinium* CSPs interactions with different nucleic acid substrates, binding to dsDNA and ssDNA probes was evaluated using *SkCSP1* and unlabeled (cold) competitors (Fig. 5). When dsDNA was used as a probe, the intensity of the slowly migrating bands decreased dramatically when the amount of competing cold ssDNA was increased. In contrast, band intensity using ssDNA probes was mostly stable using increasing amounts of cold dsDNA. Furthermore, RNA appears to compete efficiently with both dsDNA and ssDNA. These results indicate that *Sk*CSP1 has a preference for single-stranded nucleic acids, with RNA preferred over DNA. This is consistent with a previous report for *Lingulodinium* CSP1 [5]. While the potential tendency to bind to ssDNA may support a role for these proteins in uncoiling the DNA structure during transcription, preferential binding to RNA suggests this may not be their primary role.

### *L. polyedra* and *S. kawagutii* CSPs bind non-specifically to DNA and RNA

**Fig. 6:**
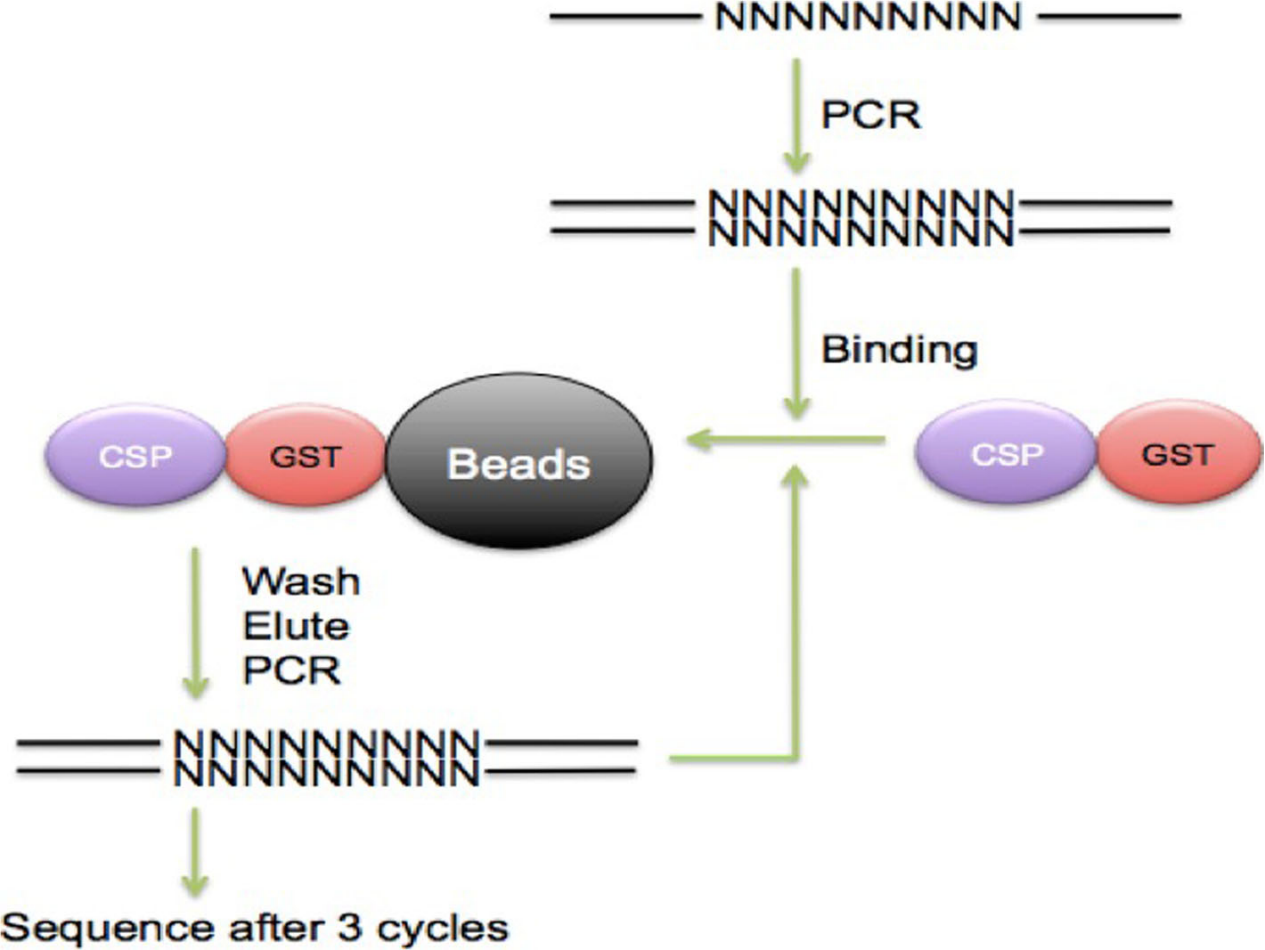
Schematic model for analyzing the specificity of DNA sequence binding by selection and amplification binding assays (SAAB)

**Fig. 7:**
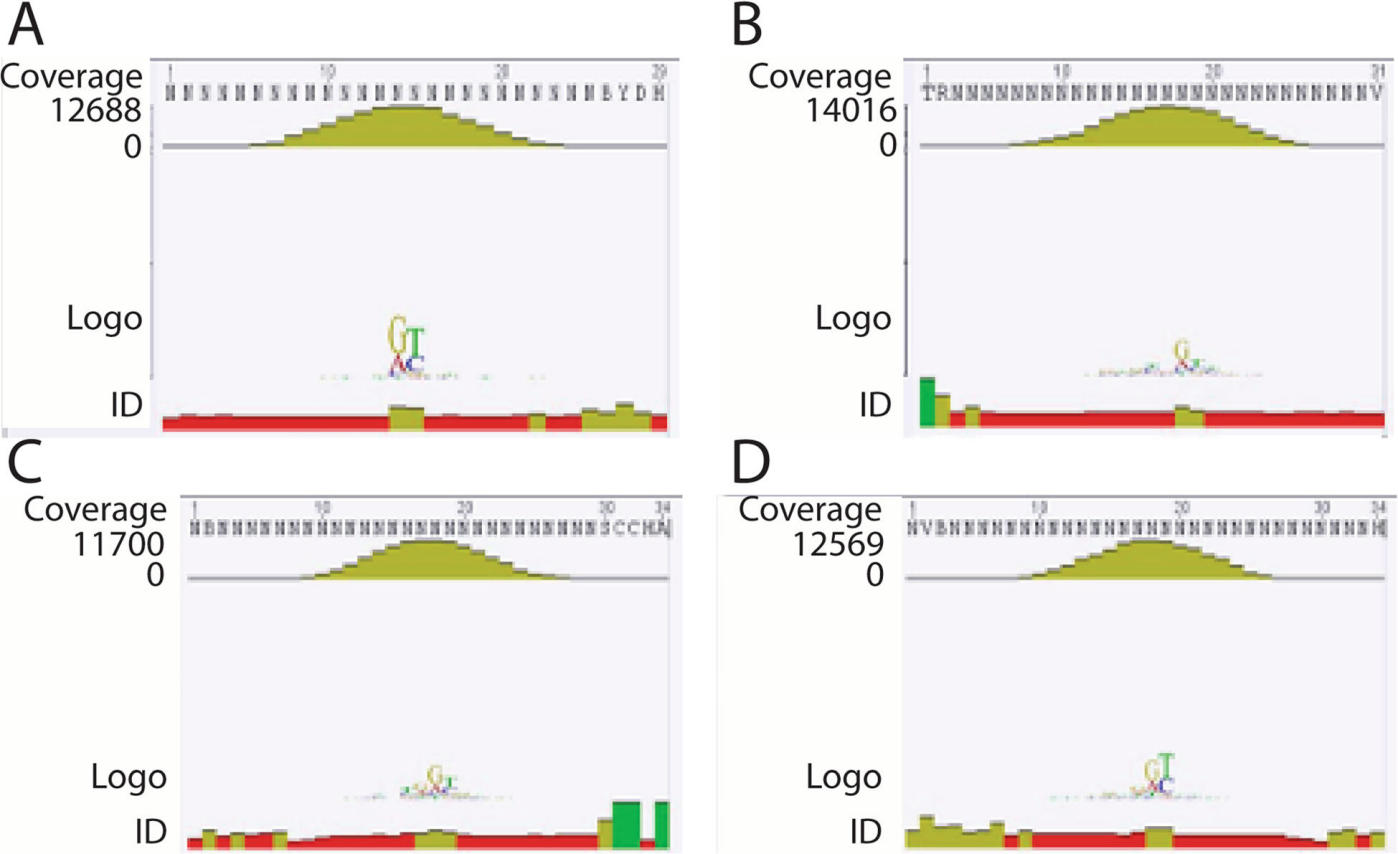
Consensus nucleotide binding activity of 4 different dinoflagellates CSPs. Over 12,000 different N9 sequences bound by *Lp*CSP1 (**a**), *Sk*CSP1 (**b**), *Sk*CSP2 (**c**), and *Sk*CSP3 (**d**) were aligned and used to prepare a sequence logo showing the frequency of each nucleotide at each position

To assess the possibility of sequence specific binding of *Lingulodinium* and *Symbiodinium* CSPs to dsDNA, we performed a selection and amplification binding enrichment (SAAB) with DNA containing 9 random nucleotides (N9) flanked by PCR primers. These experiments used the fusion proteins directly to facilitate purification of bound DNA sequences, as the presence of the GST tag did not affect DNA binding on EMSA assays. After 3 rounds of SAAB, samples containing double-stranded N9 enriched by binding to *Lp*CSP1, *Sk*CSP1, *Sk*CSP2 and *Sk*CSP3 were sequenced (Fig. 6). Over 12,000 sequences were been obtained for each CSP, but sequence alignments after binding to all four shows no evidence for a consensus motif for any of the CSPs (Fig. 7). We conclude that there is no specific dsDNA which can be enriched by binding to *Lingulodinium* or *Symbiodinium* CSPs.

## Discussion

Cold shock domain (CSD) proteins were recognized in *Escherichia coli* during cold shock stress [7, 11, 12]. The conservation of CSD in these proteins was discovered in bacteria, archaea, plants, and animals. In prokaryotes, CSPs containing only a CSD act mainly as RNA chaperones. Some *E. coli* CSPs are cold-inducible and act as RNA chaperons disrupting RNA secondary structures [7, 13]. They are also involved in the transcription regulation by binding specifically to *gyrA* promoter (CspA) [12, 14]. In eukaryotes, CSPs are composed of CSD and additional domains and aid in responding to cold stress, nutrient limitation and growth [7, 13, 15–17]. Plants CSPs are engaged in regulation of translation during cold stress and also complicated physiological processes such as seed and flower germination [7, 18, 19]. In *A. thaliana*, CSP3 interacts with other proteins involved in mRNA processing path [19]. A vertebrate CSP called YB1 (Y-box binding protein) is responsible for the regulation of transcription by binding to a Y-box specific sequence, and is also involved in regulation of translation and RNA processing [20–24] and DNA repair [7, 12, 25]. YB1 prefers to bind to ssDNA rather than dsDNA, thus disentangling the double helix structure of DNA has been proposed for the activation of transcription [7, 26]. YB1 also prefers RNA over ssDNA [7] with the consensus CA(U/C) C sequence as the RNA-binding site [27, 28]. In dinoflagellates, CSPs are mostly in the form of one conserved CSD either alone or with a C-terminal G-rich domain [5]. Previously, a Y-box sequence (CTGATTGGCT) was used to study the binding specify of *L. polyedra* CSPs [5]. Here we used different random C-rich sequences to test the possibility of sequence privileged targeting. For the SAAB assay, we synthesized a DNA sequence with 9 random nucleotides (N9) nestled between flanking PCR primers. The goal of this experiment was to see if several cycles of binding, elution and amplification would enrich for a particular sequence motif that could constitute a potential promoter element. However, no sequence motifs were enriched by binding to any of the four CSPs indicating that these proteins are unlikely to function as conventional sequence-specific transcription factors. It is not possible to rule out a role in DNA unwinding similar to what has been proposed for YB1, in which non-specific binding of CSPs to ssDNA was thought to help stabilize the structure, but it must be noted CSPs have no known helicase activity.

The importance of examining the nucleic acid binding properties of CSPs is due to the finding that the majority of the proteins annotated as transcription factors in the transcriptome of *Lingulodinium* [3], *Symbiodinium* [4] and the genome of *Symbiodinium* [10, 29] (Fig. 1) are CSDs. Our hypothesis was that to act as transcription factors, dinoflagellates CSPs should bind to dsDNA in a sequence specific manner. We assessed nucleic acid binding activity of *Lp*CSP1, *Sk*CSP1, *Sk*CSP2 and *Sk*CSP3 using two different approaches*.* In one approach, electrophoretic mobility shifts assays (EMSA) were used to show that all four CSPs could bind both double- and single- stranded DNA as well as RNA (Fig. 4). When tested in competition EMSA experiments, RNA was found to compete with binding to DNA probes better than DNA competed with binding to RNA probes (Fig. 5). These characteristics are not what would be predicted for a transcription factor. In a second approach, selection and amplification binding (SAAB) experiments showed none of the four CSPs tested enriched a specific motif after three cycles of binding and PCR amplification, again inconsistent with a role as a sequence specific transcription factor.

Our results indicate that *Lp*CSP1, *Sk*CSP1, *Sk*CSP2 and *Sk*CSP3 binding to nucleic acids does not depend on sequence. We infer that the dinoflagellate CSPs in general are unlikely to act as sequence-specific transcription factors. Although only one *S. kawagutii* CSP (*Sk*CSP1) was extensively analyzed by competition EMSA, the similarity to the *Lingulodinium* CSP1 suggests the nucleic acid binding properties found may be a consistent lineage-specific feature. The balance of the evidence thus suggests that CSPs do bind nucleic acids, thus explaining why they were annotated as transcription factors. However the details of the binding suggest they are unlikely to play this role in vivo*.* Additional characterization studies of dinoflagellate CSPs would be essential to recognize more about their function and possible interaction with other partners.

## Conclusions

The four CSPs examined here do not bind DNA in a sequence specific manner. Furthermore, *Sk*CSP1 prefers binding to single-stranded RNA. CSPs are unlikely to function as transcription factors in dinoflagellates.

## Methods

### Cell cultures

Cultures of *Symbiodinium kawagutii* (strain CCMP2468) and *Lingulodinium polyedra* (strain CCMP1936) were obtained from the National Center for Marine Algae (Boothbay Harbor, Maine). Cells were grown in f/2 sea water medium prepared from Instant Ocean under 12 h cool white fluorescent light and 12 h darkness as described [30] except that the temperature was 25 ± 1 °C for *S. kawagutii*.

### Phylogenetic reconstruction and primer design

The CSP sequences for *Lingulodinium* and *Symbiodinium* were obtained from the dinoflagellate transcriptomes deposited at NCBI and from the *Symbiodinium kawagutii* genome at the Symbiodiniaceae and Algal Genomic Resource (SAGER) database [29]. Phylogenetic analysis of CSDs from the predicted protein sequences (Table S2) was performed using a webserver for alignments (http://www.phylo.org/sub_sections/portal/) [31]. The server performs sequence alignments using MUSCLE, and curation using GBlocks. Phylogenetic reconstructions were built with RaxML using the CIPRES portal (http://www.phylo.org/sub_sections/portal/). Trees were visualized by TreeDyn. Primers were designed using Geneious software [32] or BLAST integrated into Galaxy [33] for amplification and subsequent cloning of the CSPs. Geneious software [32] was also used for sequence alignments.

### Cloning, expression and purifying of CSPs

*Symbiodinium* cultures were harvested by centrifugation and the pellets frozen in liquid nitrogen. Frozen pellets were crushed into a fine powder using a pre-chilled mortar and pestle, and the powder was added to Trizol (Invitrogen). Primer pairs based on sequences from the *Symbiodinium* transcriptome or genome were used to amplify CSPs from a first strand cDNA reaction product using the total RNA extracted from *Symbiodinium* cells as described [5]. For cDNA amplification, the reverse transcription reaction was performed with ProtoScript II first strand cDNA synthesis kit (New England BioLabs). The sequences were cloned into the pGEM-T vector (Promega) and sequenced. A second PCR was performed on the insert in the pGEM-T plasmid using primers containing restriction sites required for directional cloning into the bacterial expression vectors pGEX-4 T2 (GE Healthcare) [34] (Table. S1 in the supplementary data). The reading frame of all clones were confirmed by sequencing and the size of the CSP fusion protein verified by SDS PAGE (Fig. S1). The pGEX4T2 vectors containing CSP sequences were used to transform the chemically competent cells of BL21. Liquid Luria Bertani (LB) medium was used to grow one colony of transformed *E. coli* overnight at 37 °C with vigorous shaking in the presence of ampicillin to maintain selection for the plasmid. Protein expression were induced using Isopropyl β-D-1-thiogalactopyranoside (IPTG). Cells were collected by centrifugation, resuspended in PBS buffer and broken in a French pressure cell (Fisher Scientific). The cell lysates were then centrifuged and the supernatants were incubated with Glutathione Sepharose 4B beads (Promega) for 45 min at room temperature with end-over-end agitation. Beads were washed 4 times in PBS and resuspended in PBS supplemented with thrombin to cleave the GST tag. The size, and purity of the single CSPs were then analyzed by SDS-PAGE on acrylamide gel (Fig. S1) and the Bradford assay (BioRad) was used to assess the protein concentration.

### CSP electrophoretic mobility shift assays

[γ-^32^P] ATP (PerkinElmer) was used to 5′-end-label 32 nt ssDNA 5′-TCCGCCCTCCCTCCCCCCGCCCTCCCTCCCCA-3′ and 25 bp dsDNA 5′-GGCGCCCTCCCTCCGCCCTCCCTCA-3′ C-rich sequences using a T4 polynucleotide kinase kit (NEB). A QIAquick nucleotide removal kit (Qiagen) was used for removing the unincorporated nucleotides and purifying the probes. Either dsDNA or ssDNA 32P-labelled probes (1 ng) and increasing concentrations of CSPs (0.5–3 μg) were incubated in 20 μL of 2x binding buffer (20 mM Tris-Cl [pH 7.0], 20 mM MgCl2, 50 mM KCl, 10% glycerol and 1 mM DTT) for 30 min at room temperature. The CSP/DNA complexes were run through a 5% native polyacrylamide gels for 45 mins at 80 V in 1× Tris-borate-EDTA (TBE) buffer at room temperature. The gels were dried immediately and exposed overnight at − 80 °C with a phosphorimager screen (Amersham). The images were analyzed with a Typhoon Trio+ (Amersham) using ImageQuant 5.2. Competition reactions were prepared by incubation of the CSPs and increasing amounts of cold unlabeled ssDNA, dsDNA or RNA probes (described below) for specific binding and a 40x excess of random 22 nt single-stranded oligonucleotide (TTATTGGGGCACACCGCATGCT) for non-specific competition in the binding buffer for 15 mins before adding the radiolabeled probes.

Forty nt RNAs were synthesized by T7 RiboMAX RNA production kit (Promega) using dsDNA templates containing the N9 and T7 promoter sequences. Thereafter, RQI RNase-free DNase (Promega) was used for degradation of the dsDNA templates. The in vitro transcribed RNAs were quantitated using spectrophotometry (1.2 μg/μL), end-labeled using [γ-^32^P] ATP (PerkinElmer) (see above) and purified using filtration chromatography on a Bio-Gel P10 column (Bio-Rad). One ng labelled probe was incubated with increasing concentrations of CSPs in the binding reactions as described above.

### Selection and amplification binding assays

*Symbiodinium* and *Lingulodinium* CSPs were cloned and expressed as a fusion protein with a C-terminal GST tag as described above. The BL21 cell lysates were centrifuged and the supernatants containing GST tagged CSPs were incubated with Glutathione Sepharose 4B beads (Promega) for 45 min at room temperature with end-over-end agitation. Beads were washed 4 times in PBS*.* Immobilized *Lp*CSP1, *Sk*CSP1, *Sk*CSP2 and *Sk*CSP3 were tested for sequence-specific DNA binding activities against a set of degenerate oligonucleotides using a selection and amplification binding assay (SAAB) [35, 36]. A set of single-stranded oligonucleotides with PCR primer sequences flanking nine random nucleotides (N9) were synthesized and used to produce double-stranded DNA by a single PCR cycle using the reverse primer. Fifteen μg of double-stranded DNA (N9) was allowed to bind to 10 μL of immobilized CSPs in a 100 μL total volume solution containing 75 mM NaCl, 1 mM DTT, 1 mM phenylmethylsulfonyl fluoride, 0.1% Triton X-100, 10 ng of poly (dI-dC) per μL, 10 mM Tris-HCl (pH 7), 6% glycerol and 1% BSA. After 1 h of agitation at 4 °C, the supernatant containing unbound oligonucleotides were removed. Following 3 times of washing with binding buffer, DNA was released from the protein by boiling in water [35]. DNA was amplified in a PCR reaction to repeat the protein binding step. Three rounds of SAAB were performed before sending out the PCR products for sequencing (Fig. 6).

## Supplementary Information

**Additional file 1: Supplemental Figure S1.** Purification of LpCSP1, SkCSP1, SkCSP2 and SkCSP3. A shows recombinant LpCSP1-GST, SkCSP1-GST, SkCSP2-GST and SkCSP3-GST analyzed on an 18% acrylamide SDS-PAGE gel after affinity purification. B shows LpCSP1, SkCSP1, SkCSP2 and SkCSP3 after removal of the GST tag by thrombin digestion and binding to glutathione-Sepharose 4B beads. The sizes of the molecular weight markers (left) are shown in kilodaltons.

**Additional file 2: Supplemental Table S1.** List of primers used for PCR amplification and cloning of *LpCSP1*, *SkCSP1*, *SkCSP2* and *SkCSP3* sequences in pGEX4T2 plasmid.

**Additional file 3: Supplementary Table S2.** List of proteins selected for phylogenetic reconstruction.

## Data Availability

All data generated or analysed during this study are included in this published article.
