# Supplementary figures and images for: Assessing nucleic acid binding activity of four dinoflagellate cold shock domain proteins from Symbiodinium kawagutii and Lingulodinium polyedra
Source: BMC Mol Cell Biol. 2021 May 8;22:27. doi: 10.1186/s12860-021-00368-4 (PMC8106185; doi:10.1186/s12860-021-00368-4)

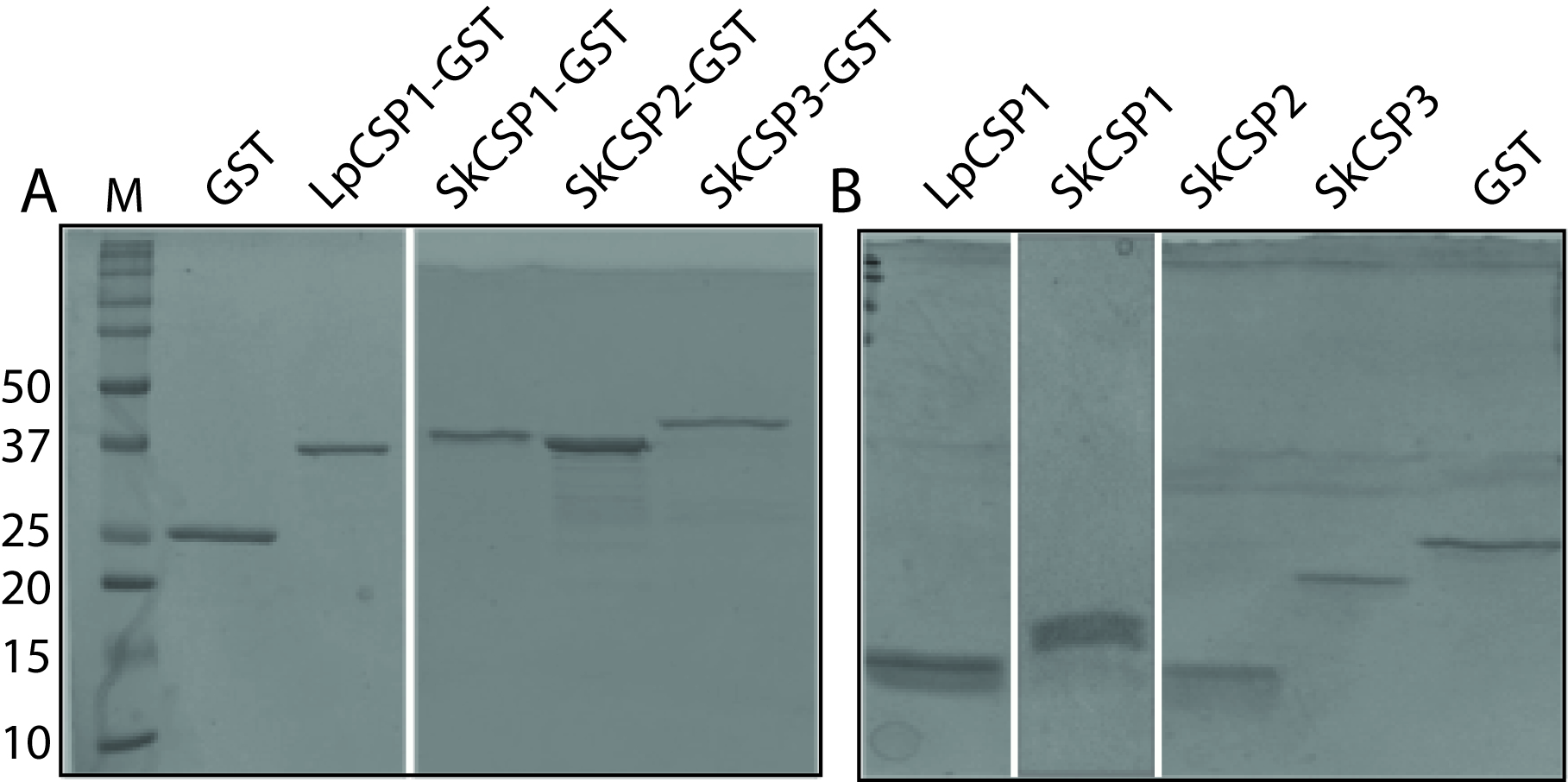

Supplement: Supplementary file 1 — Additional file 1: Supplemental Figure S1. Purification of LpCSP1, SkCSP1, SkCSP2 and SkCSP3. A shows recombinant LpCSP1-GST, SkCSP1-GST, SkCSP2-GST and SkCSP3-GST analyzed on an 18% acrylamide SDS-PAGE gel after affinity purification. B shows LpCSP1, SkCSP1, SkCSP2 and SkCSP3 after removal of the GST tag by thrombin digestion and binding to glutathione-Sepharose 4B beads. The sizes of the molecular weight markers (left) are shown in kilodaltons. [file 12860_2021_368_MOESM1_ESM.jpg]
