# Supplementary material for: Assessing nucleic acid binding activity of four dinoflagellate cold shock domain proteins from Symbiodinium kawagutii and Lingulodinium polyedra
Source: BMC Mol Cell Biol. 2021 May 8;22:27. doi: 10.1186/s12860-021-00368-4 (PMC8106185; doi:10.1186/s12860-021-00368-4)
Supplement: Supplementary file 2 — Additional file 2: Supplemental Table S1. List of primers used for PCR amplification and cloning of LpCSP1, SkCSP1, SkCSP2 and SkCSP3 sequences in pGEX4T2 plasmid. [file 12860_2021_368_MOESM2_ESM.docx]

| **Proteins** | **Accession number** | **Primers**  **name** | **Primers sequence (5’ to 3’)** |
| --- | --- | --- | --- |
| LpCSP1 | JO733348 | LpCSP1 F | gcagcaatgccttccggcactgtgaagaag |
|  |  | LpCSP1 F BamHI | tgacacGGATCCatgccttccggcactgtgaagaag |
|  |  | LpCSP1 F NdeI | TGACCATATGccttccggcactgtgaagaag |
|  |  | LpCSP1 R | accctcagctcagaaacctgaggagggtcc |
| SkCSP1 | Skav223430 | SkCSP1 F | ATGTCATATCCGAACAAATGTCGGG |
|  |  | SkCSP1 R XhoI | ATTCACTCGAGTCAGTAGGGGTCATAGCGATCAC |
|  |  | SkCSP1 F SmaI | TTGATCCCGGGATGTCATATCCGAACAAATGTCGGG |
|  |  | SkCSP1 R | TCAGTAGGGGTCATAGCGATCAC |
| SkSCP2 | Skav207008 | SkCSP2 F | ATGCCACTGGGGAAATTGAAAAA |
|  |  | SkCSP2 R XhoI | ATTCACTCGAGTCAGTCCTTGCACCAATCGG |
|  |  | SkCSP2 F SmaI | TTGATCCCGGGATGCCACTGGGGAAATTGAAAAA |
|  |  | SkCSP2 R | TCAGTCCTTGCACCAATCGG |
| SkCSP3 | Skav233957 | SkCSP3 F | ATGAATCTTCCTCCGCCTCC |
|  |  | SkCSP3 R  XhoI | ATTCACTCGAGTCAAATCATTGAGTCCTCGAAGAA |
|  |  | SkCSP3 F SmaI | TTGATCCCGGGATGAATCTTCCTCCGCCTCC |
|  |  | SkCSP3 R | TCAAATCATTGAGTCCTCGAAGAA |

**Supplemental Table S1. List of primers used for PCR amplification and cloning of *LpCSP1*, *SkCSP1*, *SkCSP2* and *SkCSP3* sequences in pGEX4T2 plasmid.**
