# Supplementary material for: Assessing nucleic acid binding activity of four dinoflagellate cold shock domain proteins from Symbiodinium kawagutii and Lingulodinium polyedra
Source: BMC Mol Cell Biol. 2021 May 8;22:27. doi: 10.1186/s12860-021-00368-4 (PMC8106185; doi:10.1186/s12860-021-00368-4)
Supplement: Supplementary file 3 — Additional file 3: Supplementary Table S2. List of proteins selected for phylogenetic reconstruction. [file 12860_2021_368_MOESM3_ESM.docx]

**Supplementary Table 2 List of proteins selected for phylogenetic reconstruction.**

| ***Alexandrium tamarense:*** | *Alexandrium tamarense* 1743629 transcribed RNA sequence GAIT01073544 |
| --- | --- |
| ***Ahrensia kielensis:*** | Cold-shock protein [*Ahrensia kielensis*] gi\|517517514\|ref\|WP_018687722.1\| |
| ***Arabidopsis* Csp1:** | Cold shock protein 1 [*Arabidopsis thaliana*] gi\|332661203\|gb\|AEE86603.1\| |
| ***Arabidopsis* Csp2:** | Glycine rich protein 2 [*Arabidopsis thaliana*] NP_195580 |
| ***Arabidopsis* Csp3:** | Cold shock domain protein 3 [*Arabidopsis thaliana*] NP_565427 |
| ***Arabidopsis* Csp4:** | Full=Cold shock domain-containing protein 4; Short=AtCSP4 Q38896 |
| ***Bos Taurus:*** | TPA: Lin-28 homolog B-like [*Bos taurus*] gi\|296484122\|tpg\|DAA26237.1\| |
| ***Cenarchaeum symbiosum:*** | Cold-shock protein [*Cenarchaeum symbiosum* A] ABK77130 |
| ***Cucumis sativus:*** | PREDICTED: Cold shock domain-containing protein 4-like [*Cucumis sativus*] gi\|449445142\|ref\|XP_004140332.1\| |
| ***Danio rerio:*** | PREDICTED: protein Lin-28 homolog A-like [*Danio rerio*] gi\|528503039\|ref\|XP_001340141.2\| |
| ***Drosophila melanogaster:*** | Lin-28 [*Drosophila melanogaster*] NP_647983 |
| ***E. coli* CspA:** | Cold shock protein CspA [*Escherichia coli* CFT073] AAN82813 |
| ***E. coli* CspB:** | CspB [*Escherichia coli*] str. K-12 AAB61739 |
| ***E. coli* CspG:** | Cold shock protein CspG NP_309172 |
| ***Falco peregrinus:*** | PREDICTED: protein Lin-28 homolog B isoform X1 [*Falco peregrinus*] gi\|529448821\|ref\|XP_005244100.1\| |
| ***Glycine max:*** | PREDICTED: glycine-rich protein 2-like [*Glycine max*] XP_003540832 |
| ***Gymnodinium catenatum:*** | Gymnodinium catenatum GcatSW0_c188 transcribed RNA sequence  GAIL01018775 |
| ***Henriciella marina***: | Cold-shock protein [*Henriciella marina*] gi\|516884417\|ref\|WP_018146825.1\| |
| ***Homo sapiens:*** | Lin-28 homolog B (*C. elegans*), isoform CRA_a [*Homo sapiens*] gi\|119568818\|gb\|EAW48433.1\| |
| ***Karenia brevis***: | K05492D08 *Karenia brevis* Multi-strain Library *Karenia brevis* cDNA 5', mRNA sequence gi\|194490792\|gb\|FK848095.1\|FK848095 |
| ***Lingulodinium* 1:** | JO733348 |
| ***Lingulodinium* 2:** | JO734870 |
| ***Lingulodinium* 3:** | JO730956 |
| ***Lingulodinium* 4:** | JO729000 |
| ***Lingulodinium* 7:** | JO766444 |
| ***Lingulodinium* 8:** | JO761018 |
| **LpCSP6 Consensus Sequence** | _ |
| ***Nicotiana tabacum:*** | Full=Glycine-rich protein 2 P27484 |
| ***Phaseolus vulgaris:*** | Hypothetical protein PHAVU_009G025100g [*Phaseolus vulgaris*] gi\|561009269\|gb\|ESW08176.1\| |
| ***Populus trichocarpa:*** | Hypothetical protein POPTR_0009s13460g [*Populus trichocarpa*] gi\|566187811\|ref\|XP_002313723.2\| |
| ***Pyrodinium bahamense:*** | TSA: *Pyrodinium bahamense* var. compressum F4W4PV301CKO5W transcribed RNA sequence gi\|509887131\|gb\|GAIO01020278.1\| |
| ***Rhizobium* CspA:** | Cold shock protein CspA [*Rhizobium leguminosarum* bv. viciae 3841] gi\|116254513\|ref\|YP_770349.1\| |
| ***Rhodopseudomonas:*** | Cold shock DNA binding protein [*Rhodopseudomonas palustris* CGA009] gi\|39936462\|ref\|NP_948738.1\| |
| ***Sinorhizobium meliloti:*** | CspA [*Sinorhizobium meliloti*] AAC64672 |
| **Skav200581 translation:** | Skav200581 [mRNA] locus=scaffold1051:18458:24228:- |
| **Skav203045 translation:** | Skav203045 [mRNA] locus=scaffold845:65342:72188:+ |
| **Skav204536 translation:** | Skav204536 [mRNA] locus=scaffold1211:212853:213298:- |
| **Skav205886 translation:** | Skav205886 [mRNA] locus=scaffold123:16538:18973:+ |
| **Skav207008 translation:** | Skav207008 [mRNA] locus=scaffold1554:61201:64400:- |
| **Skav209177 translation:** | Skav209177 [mRNA] locus=scaffold1137:469928:472431:- |
| **Skav218283 translation:** | Skav218283 [mRNA] locus=scaffold2035:589603:594139:- |
| **Skav218284 translation:** | Skav218284 [mRNA] locus=scaffold2035:596104:599782:- |
| **Skav220717 translation:** | Skav220717 [mRNA] locus=scaffold1850:102915:105042:- |
| **Skav223430 translation:** | Skav223430 [mRNA] locus=scaffold350:502771:503148:+ |
| **Skav224338 translation:** | Skav224338 [mRNA] locus=scaffold1353:319050:321488:- |
| **Skav226190 translation:** | Skav226190 [mRNA] locus=scaffold2212:105531:115729:+ |
| **Skav228973 translation:** | Skav228973 [mRNA] locus=scaffold671:194838:200215:+ |
| **Skav231215 translation:** | Skav231215 [mRNA] locus=scaffold2958:225978:226268:- |
| **Skav233957 translation:** | Skav233957 [mRNA] locus=scaffold1382:273360:273902:+ |
| **Skav234280 translation:** | Skav234280 [mRNA] locus=C9163801:2089:2301:+ |
| ***Solanum tuberosum***: | PREDICTED: glycine-rich protein 2-like [*Solanum tuberosum*] gi\|565387789\|ref\|XP_006359670.1\| |
| ***Symbiodinium:*** | TSA: *Symbiodinium* sp. clade D d_sym_30274 mRNA sequence gi\|452175549\|gb\|GAFP01006036.1\| |
